# A community–university run conference as a catalyst for addressing health disparities in an urban community

**DOI:** 10.1017/cts.2022.398

**Published:** 2022-05-06

**Authors:** Timothy F. Murphy, Rita Hubbard Robinson, Kelly M. Wofford, Alan J. Lesse, Susan Grinslade, Henry L. Taylor, Kinzer M. Pointer, George F. Nicholas, Heather Orom

**Affiliations:** 1 Community Health Equity Research Institute, Buffalo, NY, USA; 2 Jacobs School of Medicine and Biomedical Sciences, Buffalo, NY, USA; 3 Clinical and Translational Science Institute, Buffalo, NY, USA; 4 Buffalo Center for Health Equity, Buffalo, NY, USA; 5 Erie County Medical Center, Buffalo, NY, USA; 6 School of Nursing, University at Buffalo, The State University of New York, Buffalo, NY, USA; 7 School of Architecture and Planning, University at Buffalo, The State University of New York, Buffalo, NY, USA; 8 School of Public Health and Health Professions, University at Buffalo, The State University of New York, Buffalo, NY, USA

**Keywords:** Health equity, social determinants of health, conference, community engagement, health disparities

## Abstract

The African American population of Buffalo, New York experiences striking race-based health disparities due to adverse social determinants of health. A team of community leaders and university faculty determined that a community dialogue was needed to focus research and advocacy on the root causes of these disparities. In response, we organized the annual *Igniting Hope* conference series that has become the premier conference on health disparities in the region. The series, now supported by an R13 conference grant from NCATS, has been held four times (2018–2021) and has attracted community members, community leaders, university faculty, and trainees. The agenda includes talks by national leaders and breakout/working groups that led to a new state law that has reduced disproportionate traffic-ticketing and drivers' license suspensions in Black neighborhoods; mitigation of the disproportionate COVID-19 fatalities in Black communities; and the launching of a university-supported institute. We describe the key elements of success for a conference series designed by a community–university partnership to catalyze initiatives that are having an impact on social determinants of health in Buffalo.

## Introduction

Buffalo, New York, the second most populous city in the state (population 278,349), is made up of ∼50% underrepresented minorities (37% African American and 14% Hispanic), paralleling that projected for the USA in 2050. Thus, Buffalo is a microcosm of what the country will look like in 30 years. The African American Health Equity Task Force (The Task Force), formed in 2014, is a group of community stakeholders led by visionary community leaders and encompassing multiple sectors of the community, including faith leaders, community nonprofits, provider organizations, payers, government, University at Buffalo (UB) faculty, and more. The Task Force produced a comprehensive report of health indicators in African Americans in Buffalo in 2015 [1]. The report revealed striking race-based health disparities. The life expectancy of African American residents of Buffalo is five to ten years shorter compared to that of White residents of Buffalo [2]. African Americans experience a 300% increased rate of chronic diseases compared to White Buffalonians [3,4]. Much of this health disparity results from harmful social determinants of health, including poverty, underdeveloped neighborhoods, failing schools, high unemployment, low property values, poor access to public transportation, poor access to healthy food, lead contamination, and poor access to healthcare [5]. These statistics do not just reflect differences, but inequities based on injustices that stem from systemic structural racism [6,7].

The Task Force determined that a community dialogue was needed to focus research and advocacy on the root causes of health inequities in Buffalo. We took multiple approaches, including meetings with elected leaders; messaging on social media; interviews and communications with local mainstream media; a weekly talk show on health equity, “Igniting Hope Radio,” hosted by co-authors Rita Hubbard Robinson and Kelly Wofford, along with rotating leaders; and a meeting in Buffalo with leadership of the New York State Bureau of Social Determinants of Health. We also determined that a comprehensive conference with no cost to attendees would be important as part of a successful messaging campaign. Guided by this need, the annual Igniting Hope conference series was launched in 2018.

The goals of the Igniting Hope conferences were to

1. educate the community-at-large and the university community about the social determinants of health that account for race-based health disparities in Buffalo,
2. engage national experts to share their expertise and experience and to discuss and provide feedback on our work in Buffalo,
3. stimulate dialogue among community stakeholders about solutions to the adverse social determinants in our community,
4. facilitate the active participation of students from multiple disciplines to attract interested trainees into careers in health equity, and
5. leverage the conference as a springboard for initiatives to address health inequities and social determinants of health in Buffalo.

### Planning the Conferences

*Content*. Planning for the first conference began in 2017 with the formation of several subcommittees that reported to the Task Force at monthly meetings. The framework of the conferences is based on the concept of broadly engaged team science and the community engagement conference model developed by the Healthy African American Families project [8,9] in addition to elements from other successful community-academic conferences [10–13]. This model enables community voices to be heard for research to be visible and available to the community and for research results to be communicated to the community.

To educate the community-at-large and the university community about social determinants of health in Buffalo, each conference began with remarks from Task Force leaders that included a perspective on the conditions in Buffalo. To engage experts to share their expertise and to provide feedback on our work, we invited nationally recognized leaders with expertise in health disparities, racial equity, investing in communities, engaging people of color in clinical research, and historical trauma, guided by the theme of each annual conference [13]. To stimulate dialogue among community stakeholders about solutions, we included interactive breakout groups [12]. Finally, to facilitate the active participation of students from multiple disciplines, each breakout group was cofacilitated by a student and a content expert.

*Venue and timing*. Given our intention to attract community members, community leaders, university faculty, and trainees (students, interns, residents, and postdoctoral fellows), the timing and choice of venue were critical for the success of the conference. UB opened a new medical school building in November 2017 on the Buffalo Niagara Medical Campus, which is located on the East Side in a predominantly African American neighborhood. The new building was built above a subway station enabling easy access by public transportation. We viewed using this venue as an opportunity to welcome the community into the new medical school located in their neighborhood. Medical school leadership agreed to allow us to host the conference and also provided custodial and security support at their cost. After much discussion regarding day of the week, we decided on Saturday to minimize interference with job and class schedules, recognizing the risk in asking people to spend a Saturday at a conference.

Overall, the first conference was a spectacular success with 300 attendees that included community members, faculty, and a range of students from multiple disciplines, willing to devote a rainy Saturday to health disparities. Surveys were overwhelmingly positive with strong interest in an annual conference (see below). We used a similar approach for subsequent conferences with multiple subcommittees that reported to the Task Force at our monthly meetings. Each subcommittee included community members and UB faculty.

### Educating Stakeholders on Health Disparities and the Social Determinants of Health in Buffalo

A first critical step in improving health outcomes in Buffalo is to create an awareness of the scope of the problem, including the relationship of health inequities to the striking adverse social determinants of health in communities of color. Leaders of the Task Force set the stage early at each conference by reviewing selected features of race-based health disparities and social determinants of health that exist in Buffalo. Based on data from the Erie County Department of Health, African Americans in five ZIP Codes in Buffalo experience the worst health outcomes in Erie County (population 954,236) where Buffalo is located. In addition to premature mortality, they are more likely to have chronic, preventable diseases than Whites. For example, African Americans in Buffalo have a 50% higher rate of hospitalization, 25% higher deaths from heart disease, a 250% higher rate of hospitalization for diabetes, and a 500% higher rate of hospitalization for asthma compared to the White population in Buffalo [3,4]. Black males and females have a higher rate of death from all cancers than White males and females, respectively.

Buffalo is second among US cities for Black families living in poverty; 37% of Black people live in poverty compared 19% of White people in Buffalo. The Buffalo Public School System (80% Black, indigenous, and people of color) has one of the lowest high school graduation rates in the state; 14% of African Americans have a bachelor’s degree vs 36% of Whites in Buffalo. The average value of homes in Buffalo is $48,000 for Black home owners, compared to $138,000 for White home owners [5]. Most African Americans in Buffalo live in five ZIP codes on the East Side (population ∼100,000), in which there is one grocery store. The breakout groups were designed to align with the social determinants of health that are pervasive in our community.

### Engaging National Experts

The first conference was held on April 28, 2018, and was entitled “*Building a Just Community with a Culture of Health and Equity.*” The agenda included two nationally recognized keynote speakers, including Consuelo Wilkins, MD, MSCI, from Vanderbilt University and Meharry Medical College, and Stephen Thomas PhD from University of Maryland. Subsequent conferences featured nationally recognized experts whose expertise aligned with the theme of each conference (Table 1). For example, for the 2020 conference, entitled *Mobilizing Community Resources to Achieve Health Equity During a Global Pandemic,* we invited keynote speakers who focused on equity in clinical research and the devastating impact of the pandemic on communities of color. Surveys of meeting attendees consistently rated the invited keynote speakers as outstanding.

**Table 1.** Keynote speakers of Igniting Hope Conference series 2018 through 2021

Name and Affiliation	Title of Talk
**2018 *Building a Just Community with a Culture of Health and Equity***	
**Consuelo H. Wilkins, MD, MSCI** Executive Director, Meharry-Vanderbilt Alliance, Associate Professor of Medicine at Vanderbilt University School of Medicine and Meharry Medical College	*Building a Culture of Health and Equity: How It’s Done in Other Cities*
**Stephen B. Thomas, PhD** Professor, Health Policy and Management, Director, Center for Health Equity, University of Maryland School of Public Health	*Case Study: Community Impact*
**2019 *Building a Culture of Health and Ending African American Health Disparities***	
**Moro Salifu, MD, MPH, MBA, MACP** Professor and Chair of Medicine, Director of the Brooklyn Health Disparities Center, SUNY Downstate Medical Center	*Innovative Ways of Approaching Health Disparities:* *Social Determinants of Health*
**John Ruffin, PhD** Founding Director National Institute of Minority Health and Health Disparities, President and CEO of ConsulStart Inc	*The Role of Science as a Model for Understanding and Eliminating Health Disparities*
**Lisa A. Nicholas, MD, FACOG** Clinical Assistant Professor of Obstetrics and Gynecology, David Geffen School of Medicine at UCLA^1^, Los Angeles CA	*Maternal Health Disparities:* *Women’s Health and Equity*
**2020 *Mobilizing Community Resources to Achieve Health Equity During a Global Pandemic***	
**Alan J. Lesse, MD** Associate Professor of Medicine, Senior Associate Dean for Curriculum, Jacobs School of Medicine and Biomedical Sciences, University at Buffalo	*The COVID-19 Pandemic: A Magnifying Lens for Health Disparities*
**RADM Richardae Araojo, PharmD, MS** Associate Commissioner for Minority Health, Director, Office of Minority Health and Health Equity, Food and Drug Administration, Silver Spring, MD	*The FDA Perspective: Enhancing Racial and Ethnic Minority Representation in Clinical Trials*
**Raul Vazquez, MD, FAAFP** President and CEO of G-Health Enterprises, Buffalo, NY	*COVID-19, Working with the Community to Shape Better Healthcare in Light of a Pandemic*
2021 ***Healing Historical Trauma from Racist Research, Policies and Practices***	
**Thomas A. LaVeist, PhD** Dean and Weatherhead Presidential Chair in Health Equity, Tulane University School of Public Health and Tropical Health, New Orleans, LA	*My Journey to Discover Why Disparities Exist… and What to Do about It*
**Heidi L. Gartland** Chief Government and Community Relations Officer, University Hospitals, Cleveland, OH	*The Anchor Mission: Tackling Economic & Racial Disparities to Create Equitable Health Outcomes*
**Donald E. Grant, PsyD** Executive Director, Mindful Training Solutions, LLC^2^, Los Angeles, CA	*Historical Trauma: A Contemporary Conundrum*

1 UCLA: University of California Los Angeles.

2 LLC: Limited Liability Company.

### Stimulating a Dialogue Among Community Stakeholders

Breakout sessions focused on social determinants of health and were facilitated by community experts and cofacilitated by student volunteers. Attendees attended the breakout of the same topic for all three sessions because they were designed to be sequential discussions. Meeting attendees chose which breakout group in which to participate (Table 2).

**Table 2.** Themes of breakout groups of Igniting Hope Conferences 2018 through 2021

Themes of Breakout Groups
Housing
Education
Access to healthcare
Nutrition, access to healthy food, food security
Policing, fines and fees, personal security
Tobacco use
Historical trauma
Mental health
Wealth building
Restorative justice
Environment
*Impact of the pandemic on the community
*Returning to school
*COVID-19 pandemic discussion
*Employment and closing of businesses

* Topics of breakout groups at 2020 conference *Mobilizing Community Resources to Achieve Health Equity During a Global Pandemic*.

Each breakout session brought together people from widely divergent disciplines and backgrounds, an important consideration in stimulating innovative translational science advances. For example, people who work in community-based healthcare facilities, people who work in nonprofit organizations, faculty researchers, and community members interacted together in small group conversations. Few venues and opportunities exist for these types of interactions. Senior professionals interacted with early career individuals. The diversity of backgrounds of the people in these interactive sessions encouraged dialogue and sharing of experiences, facilitating productive discussions.

**Fig. 1.**
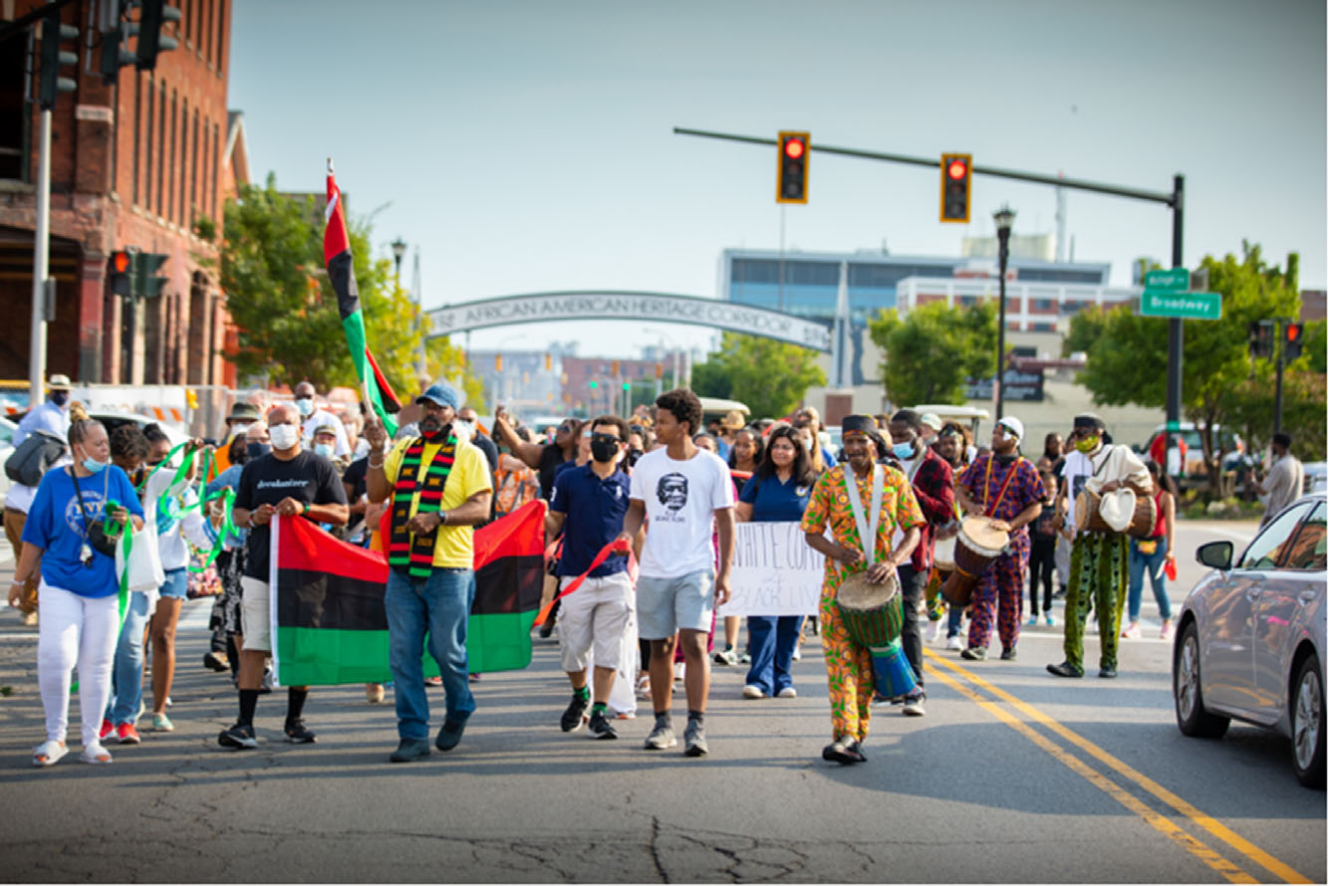
Walk of Healing leaving from the African American Heritage Corridor archway on Michigan Avenue on August 13, 2021 (Photo by Douglas Levere).

For the fourth conference in the Igniting Hope series in 2021, we introduced an innovative approach to facilitate interactions among stakeholders. We organized a two-mile “Walk of Healing” on Friday August 13 from the African American Heritage Archway and ending at the Freedom Wall, which has portraits of 28 notable civil rights leaders from America’s past and present. It was enthusiastically received. One participant commented in the evaluation survey: *“Wonderful event. I would like to see a walk of community event every year.”* A program of talks and cultural events took place at the end of the walk, which went through predominantly African American neighborhoods on the East Side. A total of 183 people took part in the walk (Fig. 1). Those who participated were inspired and energized by the event and the program.

### NIH Conference Grant and Pivoting During the Pandemic

A team of three UB faculty and three community-based leaders designed and submitted an R13 conference grant (R13 TR003486) that was funded by the National Center for Advancing Translational Sciences (NCATS) to support the Igniting Hope conferences from 2020 through 2022. In response to limitations due to the pandemic, we held the 2020 conference remotely on Saturday, August 15, 2020, and adjusted the agenda to address health equity considerations exacerbated by the pandemic with the title “*Mobilizing Community Resources to Achieve Health Equity During a Global Pandemic*.” A keynote talk was given by RADM Richardae Araojo, PharmD MS, Associate Commissioner for Minority Health, Director, Office of Minority Health and Health Equity, Food and Drug Administration, a particularly timely guest given the visibility and importance of clinical trials for safe and effective vaccines and therapeutics for COVID-19. Alan Lesse, MD, a UB infectious diseases expert and Raul Vazquez, MD, CEO of G-Health Enterprises, which provides healthcare to a large number of people in Buffalo who experience health inequities, spoke about the pandemic and its impact on the Buffalo community. The breakout group themes were relevant to peoples’ concerns during the pandemic (Table 2). In spite of the remote platform, 279 people attended the conference and the breakout discussions were active and engaging.

### Evaluation of the Conferences

Data are from participant online sign-ins, conference agendas, CTSI website analytics, and results of participant surveys. Analyses were descriptive summaries of items in the surveys. Each of the four conferences had ∼300 people attend, including the in-person conferences in 2018 and 2019 and the remote conferences in 2020 and 2021. The talks from the 2021 conference were posted and made freely available on the CTSI website. A total of 72 downloads by 44 unique viewers of the talks have been recorded in the 7 months following the 2021 conference.

For all four years of the conference, over 90% of respondents rated it “very good” or “excellent,” including 97% in 2021. Nearly all (95, 98, and 99% in years 2019–2021) said they would attend another *Igniting Hope* Conference. Over the years, the conference has routinely been described as “wonderful,” the speakers as “outstanding.” One participant remarked: *“I'm always blown away every year.”* We asked how confident they were that the conference would lead to improvements in health equity in Buffalo. The proportion who said they were very or extremely confident has generally increased from 57% to 84, 84 and 79% over the past three years suggesting that the conference and the Task Force have yielded results that instill confidence. As evidence of the cumulative impact,these conferences are having on the region, one participant commented: “*I am growing and becoming stronger in my antiracism and equity work each year.”*

Goals of the conference include providing collaboration opportunities and indeed in the 2019–2021 conferences, 96, 68%, and 81% of respondents said they made connections that could advance their work. This is a success given that the 2020 conference was entirely virtual, and 2021 conference sessions were virtual. This past year we asked researchers and trainees if the conference would change their research practices or projects; 88% said it would.

For the past two years, we assessed attendee characteristics. Approximately half were academic faculty, staff, or trainees and half were community based (Table 3). Making the conference free for attendees, including catering, and having virtual options makes it accessible to all as noted by one participant: “*Thank you for offering this conference free of charge to even those who live outside of Buffalo, NY. It was well researched and the presenters were all excellent!*”

**Table 3.** Characteristics of conference attendees who completed the evaluation survey

Attendees	2020	2021
Academic researcher, staff or trainee	59% (57)	49% (33)
Not for profit/public sector	19% (18)	22% (15)
Community member	14% (14)	19% (13)
Other	8% (8)	10% (7)
**Race/Ethnicity**		
Non-Hispanic Black	34% (31)	43% (30)
Non-Hispanic White	49% (44)	48% (34)
Hispanic	12% (11)	6% (4)
Asian	5% (4)	3% (2)
Native American or Alaska Native	−	−

### Catalytic Impact of the Conferences on the Community

Some of the breakout groups led to the formation of working groups that facilitated or contributed to tangible outcomes (Table 4). For example, a working group on nutrition and food security has been active in conducting a food bank based at a church on the East Side, organizing a conference that is bringing national experts to Buffalo in 2022 and other initiatives in various stages of development. The mental health working group organized and hosted a series of well-attended remote workshops, town halls, and support groups for community members during 2020 and 2021 with a focus on the mental health during the pandemic. The work of the fines and fees working group and the pandemic impact working group are described in more detail below.

**Table 4.** Selected working groups from Igniting Hope Conferences and outcomes to which they contributed

Working Group	Composition of group	Outcomes facilitated, including initiatives in progress
Fines and Fees Working Group	University at Buffalo (UB) School of Law faculty, community leaders, community members, Western NY Law Center, Office of State Senator Tim Kennedy	New state law passed and implemented to reduce driver’s license suspensions; reductions in traffic stops in East Side neighborhoods
Food Security Working Group	Buffalo Center for Health Equity leaders, faith-based leaders, community members	Food bank at East Side church; host national conference in Buffalo, “Building Equitable Food Systems”
Mental Health Coalition	Buffalo Center for Health Equity leaders; community-based mental health practitioners; UB faculty	Remote workshops, town halls and support groups for community members during the pandemic and beyond
Pandemic Impact Working Group	Faith-based leaders, Erie County leaders, Visiting Nursing Association of WNY, National Witness Project, UB faculty	Reduction in fatalities from COVID-19 in African Americans in Erie County
Advancing work on health equity in Buffalo	African American Health Equity Task Force leadership and UB leadership	Established UB Community Health Equity Research Institute and Buffalo Center for Health Equity, a community-based 501(c)3

*Fines and Fees Reform*. Following the 2018 Igniting Hope conference, a working group on fines and fees partnered with the Western New York Law Center to address the problem of disparate traffic ticketing in predominantly African American East Side neighborhoods resulting in suspension of a person’s driver’s license when they cannot afford to pay a traffic ticket. In 2015, the city of Buffalo implemented a plan to issue more traffic tickets and increased fines and fees. The largest increase in traffic stops between 2015 and 2019 was in the predominantly poor neighborhoods on the East Side (75.4%) and the smallest increases were in the predominantly white neighborhoods in South Buffalo (32.7%) and North Buffalo (34.1%). This practice creates a cycle of debt and punishment that especially burdens low-income communities of color. The Task Force co-hosted “community conversations” which attracted media attention and led to a series of articles in the Buffalo News and Investigative Post, raising attention to the problem at the state level. New York State Senator Tim Kennedy, who works with the Task Force, co-sponsored a bill (Senate Bill S5348B) that was passed in the 2019–2020 legislative session and signed by the governor in 2020 to reform the law related to driver’s license suspension. Changes in traffic stop practices were implemented in 2021. Community members notice that reductions in traffic stop in East Side neighborhoods and the fines and fees group is following the data. The efforts of the working group from the Igniting Hope conferences contributed to this policy change and outcome by bringing attention to the problem and engaging elected leaders.

*Mitigation of the Impact of the Pandemic on African Americans in Buffalo*. The pandemic revealed the fragility of the health and safety of African American communities in Buffalo, paralleling what is seen nationally [14–18]. On April 9, 2020, 67% of deaths in Buffalo were in African Americans, while the city’s overall population is 37% African American. In response, leaders of the Task Force and university faculty, who worked together in planning and running the first two Igniting Hope conferences, had several meetings to coordinate a comprehensive community-based effort to mitigate the disproportionate impact of the pandemic on East Side communities.

With a grant from Erie County, the Task Force worked with the Visiting Nursing Association of Western New York to bring mobile COVID-19 testing sites to the community and reached out to families to assess their needs and connected them with services and support. Call centers were established in churches to inquire about the health, social, and mental well-being and the level of food insecurity of community members. People were referred for services, connected with providers, and transportation to testing sites and doctor’s appointments were provided by the members of the National Witness Project in Buffalo. Finally, a “boots on the ground” approach was used to visit homes of those whose telephone numbers were not available. Remarkably, on May 28, 2020, 45% of deaths in Buffalo from COVID-19 were in African Americans compared to 37% of the overall population (a significant improvement over seven weeks from 67%). Buffalo is one of few communities nationally that has reduced the disparity in fatality rates in African Americans to this degree [18]. This achievement is a direct result of a strong partnership between the community and the university strengthened by continuously working together to run the Igniting Hope conferences that have led to the formation of working groups to address social determinants of health in Buffalo.

*Launch of the University at Buffalo Community Health Equity Research Institute and the community-based Buffalo Center for Health Equity*. The monthly meetings of the Task Force and the work of planning the Igniting Hope conferences led to the formation in 2019 of the community-based Buffalo Center for Health Equity (the Center) and the university-supported Community Health Equity Research Institute (the Institute). Both the university president (2019, 2020, 2021) and provost (2018, 2021) have participated in the Igniting Hope conferences, including giving welcoming remarks. This opportunity for university leadership to see the work of the Task Force first hand and the broad-based engagement of multiple sectors of community leaders through conference participation contributed to the decision to support this important new Institute that was launched in 2019 by UB [19]. It includes faculty from all 12 UB schools, enabling research by experts in many social determinants of health and is led by Timothy Murphy, MD, and four associate directors, including Rita Hubbard Robinson, JD, from the Center. The mission of the Community Health Equity Research Institute is to perform research to understand the root causes of health disparities and develop and test innovative solutions to eliminate regional health inequities.

Around the same time, the Task Force leadership launched the Buffalo Center for Health Equity, a community-based nonprofit 501(c)3 organization led by Pastor George Nicholas (Convener) and Willie Underwood, MD, MPH (Executive Director). Its goal is to end health disparities in African American communities in Buffalo. The Center and the Institute represent a landmark achievement in creating a sustainable infrastructure on which to build to address health inequities in Buffalo. We share leadership in our governance structures and work as partners, demonstrated by winning an R13 conference grant to support the 2020–2022 Igniting Hope conferences with leaders from both groups as co-investigators on the grant.

### Key Elements of Success

Review of our four-year experience reveals several key elements that were important in enabling the conference to have an impact on the social determinants of health in Buffalo. Several elements of this framework can be adapted to other communities.

The community identified the problem and initiated the effort, aligning with the Healthy African American Family conference model [8]. The comprehensive report of health indicators in African Americans in Buffalo in 2015 called attention to the problem [1]. Thus, it was the community that led the initial effort and university partners were invited to participate. This approach is fundamentally different from the traditional approach in which university faculty invite community members to contribute to a project that is faculty generated.

The conference planning was led by community members. Guided by these discussions of overall conference themes, university faculty identified and invited academic speakers and we all worked together in the many logistical components necessary to conduct a successful conference.

The broad buy-in from many sectors of the community effectively enhances the reach of the conference. To have an impact on health disparities, it will be necessary to address all the social determinants of health, the majority of which require expertise beyond the health sciences. Thus, elected leaders, business leaders, city planners, criminal justice professionals, educational leaders, and more will need to be part of the conversation. The conference series contributes toward this critical element for progress.

The conference has had the commitment of the highest levels of the university. The president and/or provost has given in-person (or remote in 2020–2021) welcomes at each conference on Saturday mornings, sending a strong message that the university is committed to the efforts toward eliminating health disparities in the Buffalo region.

An exciting component of the conferences is the active engagement and participation of students from multiple schools and disciplines. Each breakout group is cofacilitated by a student and a content expert. The student facilitator helps lead the discussion, takes notes, and then reports back to the plenary session. This approach helps influence students in their career choices, leading toward training the next generation of health disparities researchers.

Funding of the conference by an R13 conference grant from NCATS helped enhance the stature and credibility of the conference series locally and nationally. Winning a competitive NIH conference grant communicates to our local audiences (university and community) that this work is being recognized and valued nationally, which itself has a catalytic impact in attracting more contributors to the effort.

Finally, a most important outcome is the catalytic impact on working groups that implemented community interventions that have led to measurable changes in our efforts toward reducing adverse social determinants of health in Buffalo (Table 4).

Our approach embraces several principles of community engagement that can be readily adapted to other communities. The initial discussions in launching and planning the conference arose from and involved community leaders and members, creating buy-in right from the beginning. A conference design that engages students as active participants by assigning them significant roles was important in creating enthusiasm. The participation of students from multiple disciplines and from different levels of training brought diverse perspectives to the conference, adding to the academic excitement and breadth of the discussions. Engaging multiple sectors of the community in the conference is critical to reach broadly throughout the community to meaningfully address the social determinants of health. For example, the outcomes related to fines and fees and mitigating the impact of the pandemic involved engaging with governmental and community leaders. Finally, the in-person endorsement and support from the highest levels of university leadership helped to communicate that the university’s commitment to health equity is more than the effort of several committed faculty members, but rather an institutional commitment.

Our plans include conducting the annual conference with continued efforts to expand participation to include disciplines and sectors of the community that extend beyond healthcare, engaging expertise to address all social determinants of health. The Community Health Equity Research Institute is pursuing extramural funding in the form of research grants and infrastructure grants to strengthen our base of sustainability and growth. We are also committed to training the next generation of health disparities researchers through the expansion of training programs and pursuing training grants. Finally, we will continue to advance and grow our community partnerships to enhance the quality and impact of our translational research to improve the health of our community and the nation.

## References

[1] Nicholas G , Underwood W , Carter J , et al. Task Force on Health Care Disparity in the African American Community. (https://static1.squarespace.com/static/5e02325015c09a59a2d0355a/t/5e738cdeb20d7a1c1d82a7c2/1584631032813/Health+Care+Disparity+in+the+African+American+Community_Position+Paper_J.pdf).

[2] Advancing Health Equity and Inclusive Growth in Buffalo. (https://www.policylink.org/sites/default/files/BuffaloProfileFinal.pdf).

[3] Erie County Department of Health. 2017-2019 Erie County Health Assessment (2017). (http://www2.erie.gov/health/sites/www2.erie.gov.health/files/uploads/pdfs/cha.pdf).

[4] Erie County Health Indicators (2014-2016). (https://www.health.ny.gov/statistics/community/minority/county/erie.htm).

[5] University at Buffalo Regional Institute. The racial equity dividend: Buffalo’s great opportunity 2016. (https://regional-institute.buffalo.edu/wp-content/uploads/sites/155/2020/11/TheEquityDividendFINALSeptember2016.pdf).

[6] Meghani SH , Gallagher RM. Disparity vs inequity: toward reconceptualization of pain treatment disparities. Pain Medicine 2008; 9(5): 613–623. DOI 10.1111/j.1526-4637.2007.00344.x. 18777609

[7] Institute of Medicine Committee on Understanding and Eliminating Racial and Ethnic Disparities in Health Care, Smedley BD , Stith AY , Nelson AR. Unequal Treatment: Confronting Racial and Ethnic Disparities in Health Care. National Academies Press, 2003. 25032386

[8] Jones L , Collins BE. Participation in action: the Healthy African American Families community conference model. Ethnicity & Disease 2010; 20(1 Suppl 2): S2–15-20. PMC3791219 20629242

[9] Selker HP , Wilkins CH. From community engagement, to community-engaged research, to broadly engaged team science. Journal of Clinical and Translational Science 2017; 1(1): 5–6. DOI 10.1017/cts.2017.1. 31660208 PMC6798217

[10] Bharmal N , Lucas-Wright AA , Vassar SD , et al. A community engagement symposium to prevent and improve stroke outcomes in diverse communities. Progress in Community Health Partnerships: Research, Education, and Action 2016; 10(1): 149–158. DOI 10.1353/cpr.2016.0010. 27018364 PMC4943874

[11] Chan M , Fassbender K. Evaluating public engagement for a consensus development conference. Journal of Palliative Medicine Jan 2018; 21(S1): S20–S26. DOI 10.1089/jpm.2017.0390. 29283869 PMC5733652

[12] Richmond A , Aguilar-Gaxiola S , Perez-Stable EJ , et al. Proceedings of the 2017 advancing the science of community engaged research (CEnR) conference. BMC Proceedings 2019; 13(Suppl 3): 3. DOI 10.1186/s12919-019-0164-y. 31019549 PMC6474049

[13] Corpas M , Gehlenborg N , Janga SC , Bourne PE. Ten simple rules for organizing a scientific meeting. PLoS Computational Biology 2008; 4(6): e1000080. DOI 10.1371/journal.pcbi.1000080. 18584020 PMC2367436

[14] Oppel RA , Gebeloff R, Lai K K , Wright W , Smith W. The fullest look yet at the racial inequity of coronavirus. *New York Times* [Internet], 2020. (https://www.nytimes.com/interactive/2020/07/05/us/coronavirus-latinos-african-americans-cdc-data.html).

[15] Suleyman G , Fadel RA , Malette KM , et al. Clinical characteristics and morbidity associated with coronavirus disease 2019 in a series of patients in metropolitan Detroit. JAMA Network Open 2020; 3(6): e2012270. DOI 10.1001/jamanetworkopen.2020.12270. 32543702 PMC7298606

[16] Sequest TD. The disproportionate impact of COVID-19 on communities of color. NEJM Catalyst 2020. DOI 10.1056/CAT.20.0370.

[17] Price-Haywood EG , Burton J , Fort D , Seoane L. Hospitalization and mortality among Black patients and White patients with COVID-19. The New England Journal of Medicine 2020; 25(26): 2534–2543. DOI 10.1056/NEJMsa2011686. PMC7269015 32459916

[18] Mahajan UV , Larkins-Pettigrew M. Racial demographics and COVID-19 confirmed cases and deaths: a correlational analysis of 2886 US counties. Journal of Public Health 2020; 42(3): 445–447. 32435809 10.1093/pubmed/fdaa070 PMC7313814

[19] Hill DJ. New UB institute to address health disparities in Buffalo. (https://www.buffalo.edu/ubnow/stories/2019/12/community-health-equity-research-institute.html).

